# EPIDEMIOLOGICAL AND CLINICAL PROFILE OF CROHN’S DISEASE IN A BRAZILIAN REFERRAL CENTER IN 30 YEARS OF FOLLOW-UP

**DOI:** 10.1590/S0004-2803.24612025-032

**Published:** 2025-09-05

**Authors:** Igor Nolasco SEGHETO, Francisco Guilherme Cancella PENNA, Sophia Campos Salles Silva de CARVALHO, Maria de Lourdes Abreu FERRARI

**Affiliations:** 1 Universidade Federal de Minas Gerais, Belo Horizonte, MG, Brasil.; 2 Instituto Alfa de Gastroenterologia, Hospital das Clínicas, Universidade Federal de Minas Gerais, Belo Horizonte, MG, Brasil.; 3 Faculdade Ciências Médicas de Minas Gerais, Belo Horizonte, MG, Brasil.; 4 Universidade Federal de Minas Gerais, Faculdade de Medicina, Departamento de Clínica Médica, Belo Horizonte, MG, Brasil.

**Keywords:** Crohn’s disease, inflammatory bowel diseases, anti-TNF, Doença de Crohn, doenças inflamatórias intestinais, anti-TNF.

## Abstract

**Background:**

Crohn’s disease (CD) is a chronic inflammatory disease, with a heterogeneous clinical course, which can affect any segment of the gastrointestinal tract. Data on the natural history of CD in developing countries are rare.

**Objective:**

to delineate the clinical, epidemiological, and longitudinal characteristics of CD patients at a Brazilian referral center.

**Methods:**

this is an observational, cohort, retrospective study, carried out from the collection of data obtained from the medical records of individuals diagnosed with CD followed up in the period between 1991 and 2021.

**Results:**

A total of 328 participants were included, 54.3% female. The median age at diagnosis was 31 years [interquartile range (IQR)=14-45]. At diagnosis, there was a predominance of the stricturing form (38.7%) and ileocolonic location (53.7%). Among the patients with the inflammatory form, 10.8% evolved to the stricturing or penetrating forms, and the time they remained with uncomplicated disease had a median of 6 years (IQR=0-13). Aminosalicylates were used in 70.7% of the patients, but there has been a reduction in their use in the last 15 years (*P*=0.04). Corticosteroids were used in 90.2% of the participants, with a median time of use of 12 months (IQR=0-36). Immunosuppressants were used in 93.9% of participants. Two hundred and ten patients (64%) received treatment with immunobiological. The median interval between diagnosis and initiation of biological therapy was 24 months (IQR=12-60). One hundred and eighty-nine patients (57.6%) were hospitalized during follow-up, and the median hospital stay was 20 days (IQR=11-36). In the last 15 years, there was a decrease in the hospitalization rate (*P*<0.001), but there was no change in the number of hospitalizations per patient (*P*=0.62). One hundred and fifty-two patients (46.3%) underwent surgical treatment during the period evaluated and the most frequently performed surgeries were enterectomies (26.8%) and perianal procedures (25%). In the last 15 years, there has been a decrease in the rate of surgeries (*P*=0.04) and in the number of surgeries per patient (*P*<0.001).

**Conclusion:**

The data presented indicate a high prevalence of complicated CD at the onset of follow-up, alongside a significant percentage of corticosteroid use and hospitalization. However, over the past 15 years, there has been a notable reduction in hospitalization rates, surgical rates, and the number of surgeries per patient.

## HIGHLIGHTS

• Data on the natural history of CD in developing countries are rare.

• Data obtained in a Brazilian reference center in the period between 1991 and 2021.

• High rate of complicated CD at the beginning of the follow-up, as well as a high percentage of hospitalization and use of corticosteroids.

• In the last 15 years there has been a reduction in the hospitalization rate, the rate of surgeries and the number of surgeries per patient.

## RESUMO

**Contexto:**

A doença de Crohn (DC) é uma doença inflamatória crônica, de curso clínico heterogêneo, que pode acometer qualquer segmento do trato gastrointestinal. Há escassez de dados sobre a história natural da DC nos países em desenvolvimento.

**Objetivo:**

Descrever as características clínicas, epidemiológicas e evolutivas de portadores de DC em um centro de referência brasileiro.

**Métodos:**

Trata-se de estudo observacional, de coorte, retrospectivo, realizado a partir da coleta de dados obtidos em prontuários de indivíduos diagnosticados com DC, acompanhados no período entre 1991 e 2021.

**Resultados:**

Foram incluídos 328 participantes, sendo 54,3% do sexo feminino. A mediana de idade ao diagnóstico foi de 31 anos [intervalo interquartil (IQR)=14-45]. Ao diagnóstico, houve predomínio da forma estenosante (38,7%) e localização ileocolônica (53,7%). Dentre os pacientes com a forma inflamatória, 10,8% evoluíram para as formas estenosante ou penetrante, e o tempo que permaneceram com doença não complicada teve mediana de 6 anos (IQR=0-13). Os aminossalicilatos foram empregados em 70,7% dos pacientes, mas houve redução de sua utilização nos últimos 15 anos (*P*=0,04). Os corticoides foram utilizados em 90,2% dos participantes, com mediana do tempo de uso de 12 meses (IQR=0-36). Os imunossupressores foram usados em 93,9% dos participantes. Duzentos e 10 pacientes (64%) receberam tratamento com imunobiológicos. O intervalo entre o diagnóstico e o início da terapia biológica teve mediana de 24 meses (IQR=12-60). Cento e oitenta e nove pacientes (57,6%) foram hospitalizados durante o seguimento, e a mediana de permanência hospitalar foi de 20 dias (IQR=11-36). Nos últimos 15 anos houve diminuição da taxa de hospitalização (*P*<0,001), mas não houve alteração do número de hospitalização por paciente (*P*=0,62). Cento e cinquenta e dois pacientes (46,3%) foram submetidos a tratamento cirúrgico no período avaliado. Nos últimos 15 anos houve diminuição da taxa de cirurgias (*P*=0,04) e do número de cirurgias por paciente (*P*<0,001).

**Conclusão:**

Os dados apresentados mostram elevado índice de DC complicada no início do seguimento, de uso de corticoides e de hospitalização. No entanto, nos últimos 15 anos observou-se redução da taxa de hospitalização, da taxa de cirurgias e do número de cirurgias por paciente.

## INTRODUCTION

Crohn’s disease (CD) is a chronic inflammatory condition that can affect any part of the gastrointestinal tract. It exhibits a heterogeneous clinical course, varying from mild to severe forms and different phenotypes, represented by the predominance of inflammation or characterized by complications such as strictures or fistulas. The pathogenesis of CD is multifactorial, involving an interaction among genetic, epigenetic, intestinal microbiota and environmental factors. It is postulated that in individuals with genetic predisposition, intestinal cell apoptosis and the release of pro-inflammatory mediators, in conjunction with dysbiosis, altered signaling pathways, and immune dysregulation, sustain an inflammatory state that culminates in the characteristic lesions observed in the gastrointestinal tract^1^ ^,^ ^2^. 

Comprehending the natural history of CD is essential for predicting its clinical evolution and guiding therapeutic strategies. The clinical presentation of CD varies over time; although a significant proportion of patients exhibit complications at the initial diagnosis, the disease typically begins as an inflammatory condition. Over time, it may progress to more complex forms characterized by stricturing or penetrating complications. Notably, approximately one-third of patients demonstrate these complicated forms at the time of diagnosis, and the majority will develop further complications, with up to 50% requiring surgical intervention within ten years of diagnosis^3^ ^,^ ^4^.

Despite enhanced understanding of the role environmental factors play in the pathogenesis of CD, the scientific community continues to explore the reasons behind the global increase in its incidence observed over recent decades^5^ ^-^ ^10^. Nevertheless, the introduction of novel therapeutic modalities, particularly immunosuppressants and biologic therapies, has significantly contributed to the reduction of disease morbidity, resulting in improved rates of clinical and endoscopic remission in CD^11^ ^-^ ^13^. In developing countries, besides the scarcity of epidemiological data on the natural history of CD in this population, some peculiarities are observed, such as late diagnoses and treatments, often already in the complicated form of the disease, and limited availability of methods to monitor disease activity, making it difficult to decide on the choice of treatment^10^. This study aims to describe the clinical, epidemiological, and longitudinal characteristics of CD patients along 30 years, followed at a Brazilian reference center for inflammatory bowel diseases, evaluating the natural history of the disease, hospitalizations, and surgeries.

## METHODS

This was an observational, retrospective cohort study conducted through the collection of patient records after approval by the Clinical Research Ethics Committee of the Federal University of Minas Gerais - CAAE: 43254820.8.0000.5149, opinion no. 4.727.173.

The study included all individuals diagnosed with CD, who were treated at the Outpatient Clinic of the Alfa Institute of Gastroenterology at the Hospital das Clinicas of the Federal University of Minas Gerais, between 1991 and 2021, and who had attended at least one clinic visit per year during the last 5 years. The diagnosis of CD was established using Lennard-Jones criteria, based on clinical, endoscopic, histological, and radiological findings^14^. Those with less than one year of follow-up after diagnosis, unavailable medical records, incomplete data, or loss of follow-up, defined as absence of follow-up for a year or more, were excluded. The initial time considered was the moment of the first appointment at the mentioned outpatient clinic, with subsequent follow-up periods defined at one, 3, 5, 10, 15, 20, 25, and 30 years.

The following variables were analyzed: gender, age at diagnosis, time between symptom onset and diagnosis, smoking, behavior and location at year zero, presence of extraintestinal manifestations, phenotype change, presence of perianal disease, corticosteroid use, corticosteroid use at diagnosis, duration of corticosteroid use, number of corticosteroid cycles, corticosteroid dependency, corticosteroid refractoriness, immunosuppressant use, duration of immunosuppressant use, moment of onset of immunosuppressants, disease phenotype at the onset of immunosuppressants, use of biological therapy, corticosteroid-free clinical remission, endoscopic remission, rate and number of hospitalizations, length of hospital stay due to CD, rate and number of surgeries.

Patients who presented active disease despite the use of prednisolone at a dose of 1 mg/kg/day or an equivalent corticosteroid for a period of four weeks were considered corticosteroid refractory^15^. Those dependent on corticosteroids were unable to reduce the corticosteroid dose below 10 mg/day of prednisolone or equivalent within three months of its onset, without disease recurrence, or had a relapse within three months after its discontinuation^15^. Clinical remission was defined by the absence of diarrhea, abdominal pain, fever, or weight loss, equivalent to the Crohn’s Disease Activity Index (CDAI) <150^16^ and was assessed at the last consultation before data collection or at the consultation before the start of biological therapy, if used. Endoscopic remission was defined by the Simple Endoscopic Score for Crohn’s Disease (SES-CD) between 0 and 2^15^. The different CD phenotypes were defined by the Montreal Classification^17^. Early biological therapy was initiated within 18 months after diagnosis, and late if started after that period^15^ ^,^ ^18^. Regarding the strategy of initiating biological therapy, “top-down” was defined when biologics were used from the beginning of treatment, and “step-up” when biologics were initiated after conventional treatment failure with corticosteroids, aminosalicylates, and/or immunosuppressants^15^. Optimization of biological therapy was defined as the dose increase or reduction in the application intervals.

Analyses were conducted using SPSS software version 23. Normality of the distribution of numerical variables was checked by the Shapiro Wilk test. Categorical variables were expressed in terms of frequency and percentage, and numerical variables were expressed in terms of median, first, and third quartiles since they did not show a normal distribution. Comparison between categorical variables was performed using the McNemar test, and between numerical variables, the Wilcoxon test, with a significance level of 0.05.

## RESULTS

**FIGURE 1:**
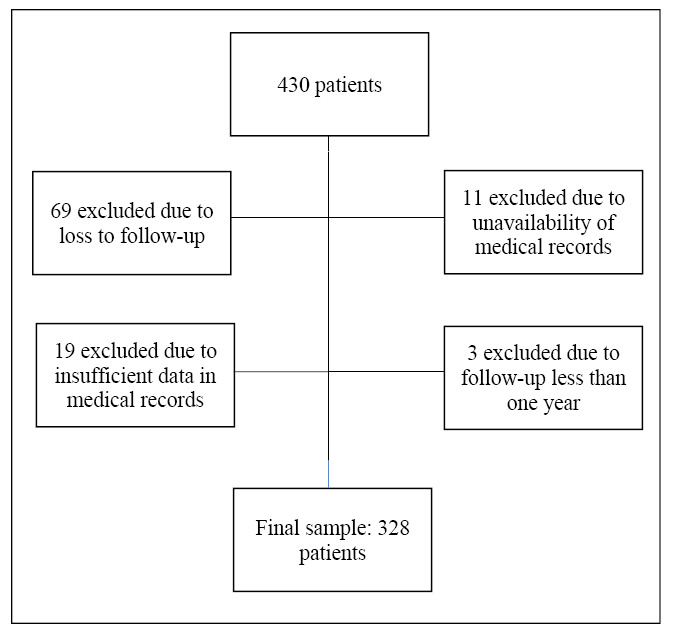
Sample universe, exclusions, and final number of study participants of patients with CD followed from 1991 to 2021.

**FIGURE 2:**
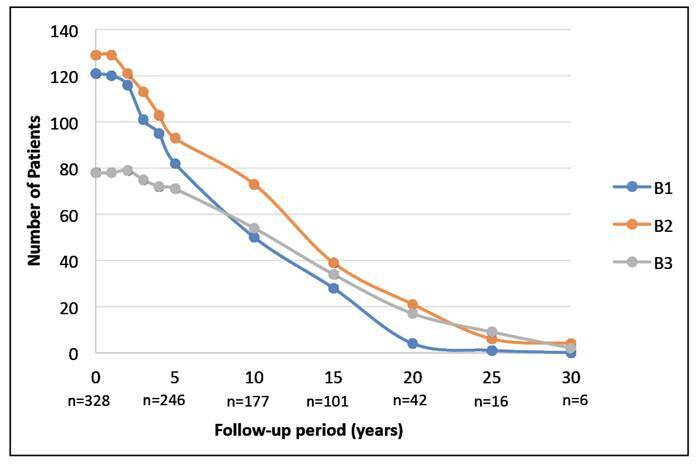
Evolution of the number of patients by phenotype over the follow-up periods from 1991 at 2021.

B1: non-stricturing and non-penetrating; B2: stricturing and B3: pene­trating^17^.

**FIGURE 3:**
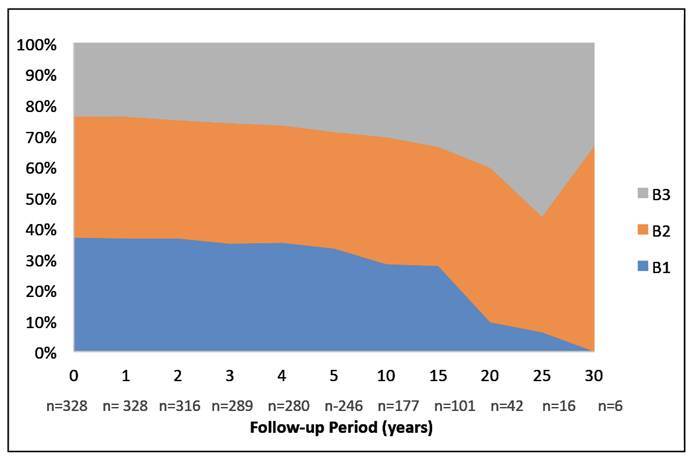
Evolution of the proportion of phenotypes over the follow-up periods from 1991 at 2021.

Note: B1: non-stricturing and non-penetrating; B2: stricturing and B3: penetrating^17^.

The sample, exclusions and final number of participants are represented in Figure 1. Medical records of 430 CD patients evaluated between 1991 and 2021 were analyzed. There were no deaths among the individuals eligible for the study. After applying the exclusion criteria, 328 participants were included, with 54.3% being female. The median follow-up time was 10 years (IQR=3-16). The median age at diagnosis was 31 years (IQR=14-45). The median time between symptom onset and diagnosis was one year (IQR=0-2). Additionally, 35% of the patients already had complications, including fistulas, abscesses, or acute abdomen at diagnosis (Table 1). Extra-intestinal manifestations were present in 31.1% of the patients over time, with the most common being joint-related: arthralgia (51%), peripheral arthritis (23%), and axial spondylarthritis (18%). Each of the following manifestations, primary sclerosing cholangitis, erythema nodosum, pyoderma gangrenosum, and venous thromboembolism, was observed in 4% of cases. Forty-seven percent of the patients had some comorbidity. Systemic arterial hypertension was the most frequent (41.5%), followed by psychiatric disorders (39.6%), among which depression (67.3%), anxiety disorders (34.5%) were noteworthy. At the onset of the follow-up period, more than half of the participants (53.7%) presented with ileocolonic location (L3), and 33.3% exhibited perianal disease. The stricturing form (B2) was the most prevalent phenotype initially, observed in 38.7% of the cases. During the follow-up period, a change in phenotype was observed in 16 patients (4.9%). These individuals maintained their initial phenotype for a median duration of six years (IQR=0-13). Among patients initially classified with non-complicated disease (B1), phenotype alterations occurred in 13 patients (10.8%). Table 1 presents the sample size and descriptive statistics. Figures 2 and 3 illustrate the progression of phenotypes within the studied population over the 30-year follow-up period.

**TABLE 1:** Demographic and clinical characteristics of the study population (n=328).

Variables	Median (1st-3rd quartile)
Age at diagnosis (years)	31 (14-45)
Interval between symptom onset and diagnosis (years)	1 (0-2)
Interval for phenotype change (years)	6 (0-13)
Variables	Descriptive - n (%)
Sex	
Female	178 (54.3)
Male	150 (45.7)
Smoking	32 (9.8)
Symptoms at diagnosis	
Diarrhea	234 (71.4)
Abdominal pain	228 (69.6)
Weight loss	136 (41.6)
Hematochezia	90 (27.3)
Fistulas, abscesses, semi-obstruction, or acute abdomen	117 (35.7)
Diagnostic criteria	
Clinical	264 (80.5)
Surgical	64 (19.5)
Extraintestinal manifestations	102 (31.1)
Location in year 0	
L1	92 (28.1)
L2	46 (14.0)
L3	176 (53.7)
L4	8 (2.4)
L1+L4	3 (0.9)
L3+L4	3 (0.9)
Behavior in year 0	
B1	120 (36.6)
B2	127 (38.7)
B3	81 (24.7)
Presence of perianal disease	
No	222 (67.7)
At diagnosis	73 (22.3)
After diagnosis	33 (10.0)
Phenotype change	
No	312 (95.2)
Yes	16 (4.8)
New phenotype	
B2	9 (56.2)
B3	7 (43.8)

L1: ileum; L2: colonic; L3: ileocolonic; L4: high TGI; B1: non-stricturing/non-penetrating; B2: stricturing; B3: penetrating^17^.

In the management of CD, aminosalicylates were administered to 70.7% of the patients. From 1991 to 2005, 82% of patients received aminosalicylates, compared to 68% between 2006 and 2021 (*P*=0.04). Corticosteroids were utilized in 90.2% of cases. Corticosteroid use was reported at 95% between 1991 and 2005, and 89% between 2006 and 2021 (*P*=0.23). Immunosuppressants were prescribed to 93.9% of participants; among them, 81.4% commenced therapy within the first two years of follow-up. Table 2 describes in detail the statistics on the treatment of the studied population.

**TABLE 2:** Descriptive analysis of conventional treatment in the population studied between 1991 and 2021 (n=328).

Variable	Descriptive- n (%)
Use of aminossalicylates	232 (70.7)
Use of corticosteroids	296 (90.2)
Use of corticosteroids at diagnosis	
No	50 (9.8)
Yes	253 (77.1)
Unknown	25 (7.6)
Numbers of corticosteroid cycles	
0	32 (9.8)
1	139 (42.4)
2	71 (21.6)
3 or more	68 (20.7)
Unknown	18 (5.5)
Corticosteroid dependence	136 (41.5)
Corticosteroid refractoriness	27 (8.2)
Use of immunosuppressants	308 (93.9)
Time of onset of immunosuppressants	
Year 0	211 (68.5)
Year 1	30 (9.7)
Year 2	10 (3.2)
Year 3 or later	57 (18.6)
Variable	Median (1st-3rd quartile)
Duration of corticosteroid use (months)	12 (0-36)

Two hundred and ten participants (64%) underwent treatment with biologics. Anti-TNF agents were utilized in 99.5% of these patients, and a ‘step-up’ strategy was employed in approximately 95% of cases. The median time from diagnosis to the initiation of biologic therapy was 24 months (IQR=12-60). Early therapy, initiated within the first 18 months post-diagnosis, was administered to 71 patients (33.8%), with the majority (78.9%) starting after 2013. Infliximab was the first biologic administered in 62.9% of cases, followed by adalimumab in 34.3%. Biologics were used in combination with immunosuppressants in 86% of cases, and as monotherapy in 14%.

Among patients who used infliximab as their first biologic, its use was combined with immunosuppressants in 92.5% of cases. Within this subgroup, optimization was required in 24% of patients, and the median interval between the start of biologic therapy and the moment of optimization was 38 months (IQR=18-68). A switch in biologic was necessary in 30 patients (22%) of this subgroup, with six due to non-response, 20 due to loss of response, and four due to treatment-related complications. Among patients for whom adalimumab was the first choice, 22% used it as monotherapy and 78% in combination with immunosuppressants. In this subgroup, optimization was required in 33.3% of patients, and the median interval between the start of biologic therapy and the moment of optimization was 18 months (IQR=8-48). A switch in biologic therapy was necessary in 18 patients (25%) of this subgroup, with eight due to non-response, eight due to loss of response, and two due to treatment-related complications.

With conventional treatment, the clinical remission rate was 31.7%, and the endoscopic remission rate, evaluated in a total of 260 patients who underwent colonoscopy for follow-up, was 20.4%. In a total of 210 patients undergoing biologic therapy, 166 had endoscopic response assessed, with remission found in 43.9% of cases. The clinical remission rate was 67.6% after the use of biologics. Table 3 presents descriptive statistics related to the use of biological therapy in the population studied.

**TABLE 3:** Characteristics of the 210 patients treated with biological therapy from 1991 at 2021.

Variables	Descriptive- n (%)
First biologic therapy	
Infliximabe	132 (62.9)
Adalimumabe	72 (34.3)
Certolizumabe	5 (2.3)
Ustequinumabe	1 (0.5)
Time of initiation of biologic therapy	
≤18 months from diagnosis	71 (33.8)
>18 months from diagnosis	139 (66.2)
Indication of biologic therapy	
Non-response to conventional treatment	128 (61.0)
Severe disease with worse clinical prognosis criteria	46 (21.9)
Presence of perianal disease	34 (16.2)
Prevention of postoperative recurrence	2 (1.0)
Behavior at the start of biological therapy	
B1	71 (33.8)
B2	81 (38.6)
B3	58 (27.6)
Need for optimization of biologic therapy	56 (26.7)
Biologic therapy switch	48 (22.9)
Reason for immunobiological change (n=48)	
Loss of response	28 (58.3)
Lack of response	12 (25.0)
Adverse effect	6 (12.5)
Treatment complications (opportunistic infections)	2 (4.1)
Second biologic (n=48)	
Adalimumabe	26 (54.2)
Infliximabe	17 (35.4)
Ustequinumabe	3 (6.3)
Certolizumabe	1 (2.1)
Vedolizumabe	1 (2.1)
Adverse effect of biologic therapy (n=6)	
Autoimmune hepatitis	2 (33.3)
Skin lesions (psoriasis)	2 (33.3)
Acute pancreatitis	1 (16.6)
Anaphylaxis	1 (16.6)
Variables	Median (1st-3rd quartile)
Interval between diagnosis and initiation of biologic therapy (months)	24 (12-60)
Interval between initiation of biologic and optimization (months)	24 (4-71)

B1: non-stricturing/non-penetrating; B2: stricturing; B3: penetrating^17^.

During the follow-up period, 189 patients (57.6%) required hospitalization. In those hospitalized due to CD, the median total length of hospital stay was 20 days (IQR=11-36). During the evaluated period, 152 patients (46.3%) underwent surgical procedures related to CD. Of these, 35% required two or more surgeries. Table 4 provides a detailed description of hospitalizations and surgeries performed during the study period.

**TABLE 4:** Descriptive analysis of hospitalizations and surgeries in the population studied between 1991 and 2021 (n=328).

Variables	Descriptive- n (%)
Hospitalization	
No	139 (42.4)
Yes	189 (57.6)
Number of Hospitalizations	
1	87 (26.5)
2 or more	102 (31.0)
Total Hospitalizations	
Clinic	276 (73.0)
Surgical	102 (27.0)
Clinical hospitalization (indication)	
Disease activity	152 (55.2)
Complications due to the disease	115 (41.7)
Complications arising from treatment	9 (3.1)
Surgical hospitalization (indication)	
Acute abdomen	72 (70.5)
Fistulas or abscesses	20 (19.6)
Elective surgeries	10 (9.9)
Surgeries	
No	176 (53.7)
Yes	152 (46.3)
Number of surgeries	
1	99 (30.1)
2	40 (12.2)
3 or more	13 (4.0)
Total surgeries	
Abdominal surgeries	168 (75.0)
Perianal surgeries	56 (25.0)
Variables	Median (1st-3rd quartile)
Number of days in hospital	20 (11-36)

**FIGURE 4:**
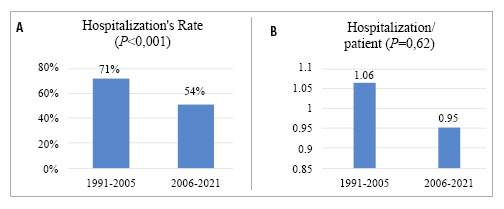
A/B. Evolution of hospitalizations between 1991 and 2021.

Between 1991 and 2005, the hospitalization rate was 71% and in the number of hospitalizations per patient was 1.06. Between 2006 and 2021, the hospitalization rate was 54% while the number of hospitalizations per patient was 0.95. The difference in hospitalization rates between the two periods was statistically significant (*P*<0.001); however, there was no significant difference in the number of hospitalizations per patient (*P*=0.62). Figures 4A and 4B illustrate the evolution of hospitalization over the 30-year follow-up.

**FIGURE 5:**
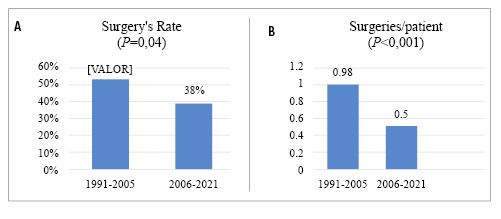
A/B. Evolution of surgeries between 1991 and 2021.

In the period between 1991 and 2005, 60 surgeries were performed, averaging 0.98 surgeries per patient, with a surgical rate of 52%. In contrast, from 2006 to 2021, the surgical rate decreased to 38%, and the number of surgeries per patient reduced to 0.5. Figures 5A and 5B illustrate the evolution of surgical interventions in the cohort over the 30-year follow-up. The differences in surgery rates (*P*=0.04) and the number of surgeries per patient (*P*<0.001) between the two periods were statistically significant. 

## DISCUSSION

In this study, most patients presented with the complicated form of CD at diagnosis, while approximately one-third exhibited the B1 behavior. These findings contrast with both national and international studies, which typically report a predominance of the inflammatory phenotype at diagnosis^19^ ^-^ ^21^. A cohort on the natural history of CD demonstrated that at the time of diagnosis, 56 to 81% of patients had non-stricturing and non-penetrating behavior^3^. Additionally, another study indicated that individuals with B1 behavior face a 51% risk of developing complications within 20 years post-diagnosis^22^. The predominance of the complicated form of CD in the studied population could be justified by the fact that the study was conducted at a public healthcare IBD reference center in Brazil, which patients with greater severity are referred, or less likely it may reflect the disease profile in this region of the country.

Regarding the time between the onset of symptoms and diagnosis, studies have shown that delayed diagnosis correlates with an increased risk of complications, such as strictures, fistulas, and surgeries^23^ ^-^ ^24^. Despite the predominance of complicated disease phenotypes at diagnosis, the interval between the onset of symptoms and diagnosis in this study was shorter than those reported in other Brazilian studies. This variance may be attributed to the intermittent nature of CD and potential recall bias among patients, which could affect the accuracy of symptom onset reporting. For instance, a retrospective study conducted in Piaui, a Northeastern state of Brazil, reported an average delay of 35.5 months in CD diagnosis. Conversely, a retrospective study in São Paulo, a Southeastern state, observed a diagnostic delay of 20 months (range 6-48 months)^19^ ^,^ ^20^. On the other hand, European and Asian studies, although heterogeneous, have shown a mean delay in CD diagnosis of less than 12 months^25^ ^-^ ^27^.

The data described showed a high rate of aminosalicylate and corticosteroid use; however, there was a reduction in aminosalicylate use from 2006, which was not observed concerning corticosteroids. In another Brazilian study published in 2021, salicylates were used in 88.1% of patients^19^. Despite the decrease in usage observed in the last decade, mainly due to the increased availability of immunosuppressants and biologics, and restrictions on indications according to the CD management guidelines, a high percentage of aminosalicylate use is still observed in Brazil^15^ ^,^ ^21^ ^,^ ^28^. Another contrast observed in CD treatment in developing countries is the high rate of corticosteroid use and dependence. Studies in Latin America show corticosteroid usage rates above 75%^8^ ^,^ ^19^. In contrast, a study conducted in the North American population showed that 43% of CD patients received corticosteroids for treatment, and 28% were considered corticosteroid dependent^29^. The difficulty of access to immunomodulators and biologics helps explain the high rate of corticosteroid usage in Brazil, a drug that remains a cornerstone for the treatment of active disease but is ineffective in maintaining remission^15^.

Immunosuppressors and immunobiologics were also used more frequently in the presented sample than described in the literature. According to Kotze et al., the use of immunomodulators in Brazil increased from 8.3% between 1970-1998 to 71.7% in 2013-2014, and the use of anti-TNF increased from 29.6% between 2005 and 2012 to 43.4% in 2013-2014^8^. In a meta-analysis of 198 population-based studies involving Europe, Latin America, and Asia, immunomodulators and biologics were used in 32-60% and 6-20% of CD patients, respectively^30^. A study conducted in the same hospital of this study, before 2012, showed that immunosuppressants were used in 76.4% of patients, and biologics in 26.4%^28^. The high percentage of biologic usage in the studied population can be explained by the predominance of complicated disease phenotypes (Montreal classifications B2 or B3)^17^ and a high rate of perianal disease, known factors of worse prognosis. The high rate of immunosuppressant usage can be justified by the clinical protocol and therapeutic guidelines (PCDT) for CD in Brazil, which establishes the requirement for failure of conventional treatment with immunomodulators before allowing biologic therapy. This also underlies the predominance of the ‘step-up’ strategy over the ‘top-down’ strategy in the presented data.

The hospitalization rate described over 30 years of follow-up is high compared to developed countries; however, a decrease in hospitalization was observed in the last 15 years. This change in recent years is probably due to better clinical and endoscopic remission rates achieved with the use of immunosuppressors and biologics. Scientific evidence demonstrates that mucosal healing, the goal of this therapy, correlates with an increased rate of clinical remission free of corticosteroids, lower hospitalization and surgery rates, and an improvement in patients’ quality of life^11^ ^-^ ^13^. An article published in 1994 on a cohort in Denmark, in a context of limited treatment options, showed that 83% of patients were hospitalized in the first year of the disease^31^. In another study conducted in the pre-biologic era in the United States, it was found that the cumulative risk of hospitalization within 10 years of the disease was 63%^32^. In a systematic review published in 2020, which included 12 cohorts from six different countries, a hospitalization rate of 23.3% was observed^33^. In Brazil, a retrospective study revealed a 24% reduction in the hospitalization rate between 2005 and 2015, and a systematic review showed a reduction in CD-related hospitalizations from 83.3% between 1980-1999 to 29.2% in 2006^8^ ^,^ ^34^.

The observed surgery rate in the present case series was like that described in the literature. Like hospitalizations, a decrease in the surgery rate and the number of surgeries per patient was observed in the last 15 years. A population-based study conducted in Germany showed that, despite the development of new therapies, the number of patients requiring surgery for CD remained stable between 2010 and 2017, and despite the trend of less invasive surgeries, no decrease in the overall complication rate was observed^35^. However, other studies have shown that the use of immunosuppressants and biologic therapy has contributed to a reduced need for CD-related surgeries. A systematic review published by Frolkis et al. showed that the cumulative incidence of surgery at 10 years of disease was 46.6% but was lower than 40% for patients diagnosed after 1980^36^. Another systematic review estimated the risk of a second surgery in CD at 35%, and similarly, this risk was significantly lower among patients diagnosed after 1980^37^.

In the presented case series, the median age at diagnosis is consistent with the A2 phenotype of the Montreal Classification^17^, described as the most common in most studies, and the predominance of females is also seen in other national and international studies^5^ ^,^ ^9^ ^,^ ^19^. The smoking rate shows a decreasing trend among CD patients followed at our institution in recent years. A study analyzing the smoking rate in these patients before 2012 revealed that 21.7% were smokers, higher than what was observed in the present study^28^. Regarding the disease location, it is observed that approximately 50% of CD patients have ileocolitis, and about 80% have small bowel involvement, usually the terminal ileum^18^. Similarly, in the studied population, there was a predominance of ileocolonic location, and more than two-thirds of patients were involved of the terminal ileum. Other studies in Brazil shown disparate results regarding CD location. A study analyzing the clinical and demographic profile at a reference center in São Paulo, Southeastern Brazil, revealed that 47.9% had ileocolonic disease^19^. Another study in Northeastern Brazil showed a predominance of colonic location^20^. A systematic review that assessed IBD in Latin America and the Caribbean demonstrated that, despite heterogeneous data, the predominant phenotypic profile was diagnosis age between 17 and 40 years (Montreal Classification A2), ileocolonic location (L3), and non-stricturing and non-penetrating behavior (B1)^8^ ^,^ ^17^.

In the present study, the most common symptoms at diagnosis were diarrhea, abdominal pain, and weight loss. According to the literature, chronic diarrhea is the cardinal symptom for CD diagnosis, and abdominal pain and weight loss are observed in about 80% and 60% of cases, respectively^18^ ^,^ ^38^. Up to 50% of IBD patients experience at least one extraintestinal manifestation (EIM) during their lifetime, and of these, 10 to 35% present with inflammatory arthropathies^39^, like to what was observed in the studied population. Other studies in Brazil shown heterogeneous results regarding the prevalence of EIM, with results ranging from 22.3% to 78.2%^19^ ^,^ ^28^. Another point to highlight is the high prevalence of mental disorders among individuals diagnosed with CD, with mood disorders and generalized anxiety disorders being the most common. In recent years, articles have been published reinforcing this association, highlighting that the prevalence among IBD patients is higher than in the general population, which can negatively impact treatment and result in a poorer quality of life for patients^40^ ^,^ ^41^.

Despite the relevance of the data presented, it should be noted that the study has limitations inherent to the retrospective design of the study and being carried out at a single center. The unavailability of data on hospitalizations, surgeries, and treatments performed at other facilities, as well as incomplete medical records, were also limitations encountered. The Outpatient Clinic of the Alfa Gastroenterology Institute at the Hospital das Clinicas of the Federal University of Minas Gerais is a regional reference center for IBD treatment, to which patients with greater severity are referred. This can make it difficult to evaluate the evolution of uncomplicated forms of the disease to complicated forms; however, this peculiarity allows an analysis of the natural history of CD in this patient profile.

## CONCLUSION

The data presented indicate high rates of complicated CD at the beginning of the follow-up. There was also a high percentage of aminosalicylate and corticosteroid use, as well as hospitalizations. Over the last 15 years, there has been a decrease in hospitalizations, surgeries, and the number of surgeries per patient. The high rate of complicated disease in the case series posed a challenge in assessing the impact of treatment on the natural history of the disease. However, this peculiarity allowed an evaluation of the influence of immunosuppressants and biologics on the progression of CD, even in this profile of more severe patients.
